# Global lactylome reveals lactylation-dependent mechanisms underlying T_H_17 differentiation in experimental autoimmune uveitis

**DOI:** 10.1126/sciadv.adh4655

**Published:** 2023-10-18

**Authors:** Wei Fan, Xiaotang Wang, Shuhao Zeng, Na Li, Guoqing Wang, Ruonan Li, Siyuan He, Wanqian Li, Jiaxing Huang, Xingran Li, Jiangyi Liu, Shengping Hou

**Affiliations:** ^1^ The First Affiliated Hospital of Chongqing Medical University, Chongqing, China.; ^2^ Chongqing Key Laboratory of Ophthalmology, Chongqing, China.; ^3^ Chongqing Eye Institute, Chongqing, China.; ^4^ Chongqing Branch of National Clinical Research Center for Ocular Diseases, Chongqing, China.; ^5^ School of Basic Medical Sciences, Chongqing Medical University, Chongqing 400016, China.; ^6^ Beijing Institute of Ophthalmology, Beijing Tongren Eye Center, Beijing Tongren Hospital, Capital Medical University, Beijing Ophthalmology & Visual Sciences Key Laboratory, Beijing, 100730, China.

## Abstract

Dysregulation of CD4^+^ T cell differentiation is linked to autoimmune diseases. Metabolic reprogramming from oxidative phosphorylation to glycolysis and accumulation of lactate are involved in this process. However, the underlying mechanisms remain unclear. Our study showed that lactate-derived lactylation regulated CD4^+^ T cell differentiation. Lactylation levels in CD4^+^ T cells increased with the progression of experimental autoimmune uveitis (EAU). Inhibition of lactylation suppressed T_H_17 differentiation and attenuated EAU inflammation. The global lactylome revealed the landscape of lactylated sites and proteins in the CD4^+^ T cells of normal and EAU mice. Specifically, hyperlactylation of Ikzf1 at Lys^164^ promoted T_H_17 differentiation by directly modulating the expression of T_H_17-related genes, including Runx1, Tlr4, interleukin-2 (IL-2), and IL-4. Delactylation of Ikzf1 at Lys^164^ impaired T_H_17 differentiation. These findings exemplify how glycolysis regulates the site specificity of protein lactylation to promote T_H_17 differentiation and implicate Ikzf1 lactylation as a potential therapeutic target for autoimmune diseases.

## Teaser

Lactylation levels of Ikzf1 at lys164 were elevated in CD4^+^ T cells of EAU mice and regulated T_H_17 differentiation.

## INTRODUCTION

The differentiation of naïve CD4^+^ T cells into subtypes with different functions is important in immune defense, but dysregulation of this process can induce multiple inflammatory and autoimmune diseases, including autoimmune uveitis (*1*, *2*), systemic lupus erythematosus (*3*), multiple sclerosis (*4*), and Crohn’s disease (*5*). Effector CD4^+^ T cells, particularly interferon-γ (IFN-γ) producing T helper 1 (T_H_1) cells and interleukin-17 (IL-17) producing T_H_17 cells, are the major immunopathogenic cells that contribute to the development of autoimmune disorders. Regulatory T cells (T_regs_), which are CD4^+^FoxP3^+^CD25^+^, inhibit the activity of effector CD4^+^ T cells and support immunological homeostasis. The balance between effector CD4^+^ T cells and T_regs_ is essential for the emergence of autoimmune diseases (*6*, *7*). Elucidating the mechanisms underlying the modulation of CD4^+^ T cell differentiation is important to develop targeted therapies.

Dynamic metabolic reprogramming from oxidative phosphorylation to glycolysis plays important roles in CD4^+^ T cell activation and differentiation (*8*–*11*). In response to activation, effector T cells undergo aerobic glycolysis to promote rapid adenosine triphosphate (ATP) synthesis (*11*). Lactate is an end product of glycolysis and one of the most enriched by-products of metabolism in inflamed tissue microenvironments (*8*, *11*). Lactate content is elevated in the synovia of patients with rheumatoid arthritis (*12*) and in the sera of patients with multiple sclerosis and Sjogren’s syndrome (*13*–*15*). Inhibition of glycolysis or ablation of lactate dehydrogenase A in T cells impairs the activation, proliferation, and differentiation of T cells and diminishes pathological immune responses in autoimmune diseases (*11*, *16*). However, the mechanisms by which lactate accumulation affects CD4^+^ T cell differentiation are largely unclear.

Lactate influences cellular processes by forming lysine lactylation (Kla) in certain protein residues and affecting protein functions (*17*–*20*). Lactylation is a recently described posttranslational modification. Accumulating evidence suggests that lactylation plays an important role in transducing metabolic changes into stable gene expression patterns. Increased lactylation of histone proteins can directly stimulate gene transcription and influence macrophage polarization (*17*), microglial inflammation (*18*), and tumorigenesis (*19*). It can also modulate the functions of nonhistone proteins. For example, lactylation of MOESIN induces its interaction with the transforming growth factor–β (TGF-β) receptor and promotes tumorigenesis (*20*). We previously showed that lactylation of YY1 in retinal microglia promotes retinal neovascularization (*21*). However, whether and how up-regulated glycolysis modulates CD4^+^ T cell differentiation via lactylation remains obscure.

Ikzf1, a member of the Ikaros transcription factor family, plays important roles in regulating lymphocyte development (*22*). Ikzf1 is essential for T_H_17 differentiation. Naïve CD4^+^ T cells cannot differentiate into T_H_17 cells without Ikzf1 (*23*). Meanwhile, mutations in Ikzf1 have been associated with various inflammatory and immune system diseases, including acute lymphoblastic leukemia, immunoglobulin A (IgA) vasculitis, and systemic lupus erythematosus (*24*–*27*). However, the specific mechanisms by which Ikzf1 modulates T_H_17 differentiation remain to be determined. Experimental autoimmune uveitis (EAU) is a widely accepted animal model for noninfectious uveitis. Progression of EAU is induced by dysregulation of effector T cells, and studies of this animal model have gradually revealed the potential pathogenic roles of autoimmune disorders in uveitis (*1*, *28*).

In the current study, we explored the role of lactylation in modulating CD4^+^ T cell differentiation. We found that the lactylation levels in CD4^+^ T cells increased with EAU progression. Suppressing lactylation by using glycolysis inhibitors prevented T_H_17 differentiation and ameliorated EAU progression. Moreover, we characterized the entire landscape of differentially lactylated sites and proteins between normal and EAU CD4^+^ T cells. Further experiments showed that the lactylation levels of Ikzf1at Lys^164^ increased in the CD4^+^ T cells of EAU mice and regulated T_H_17 differentiation. CUT& Tag analysis indicated that the lactylation of Ikzf1 regulated its binding to the promoters of T_H_17-related genes, including runt-related transcription factor (Runx1), Toll-like receptor 4 (Tlr4), IL-2, and IL-4 and differentially modulated the expression of these genes. Mutation at this lactylated site impaired T_H_17 differentiation. Overall, we propose an important function of lactylation in CD4^+^ T cell differentiation and provide insights into the pathogenesis of autoimmune uveitis.

## RESULTS

### Elevated lactylation levels in CD4^+^ T cells of EAU mice

**Fig. 1.**
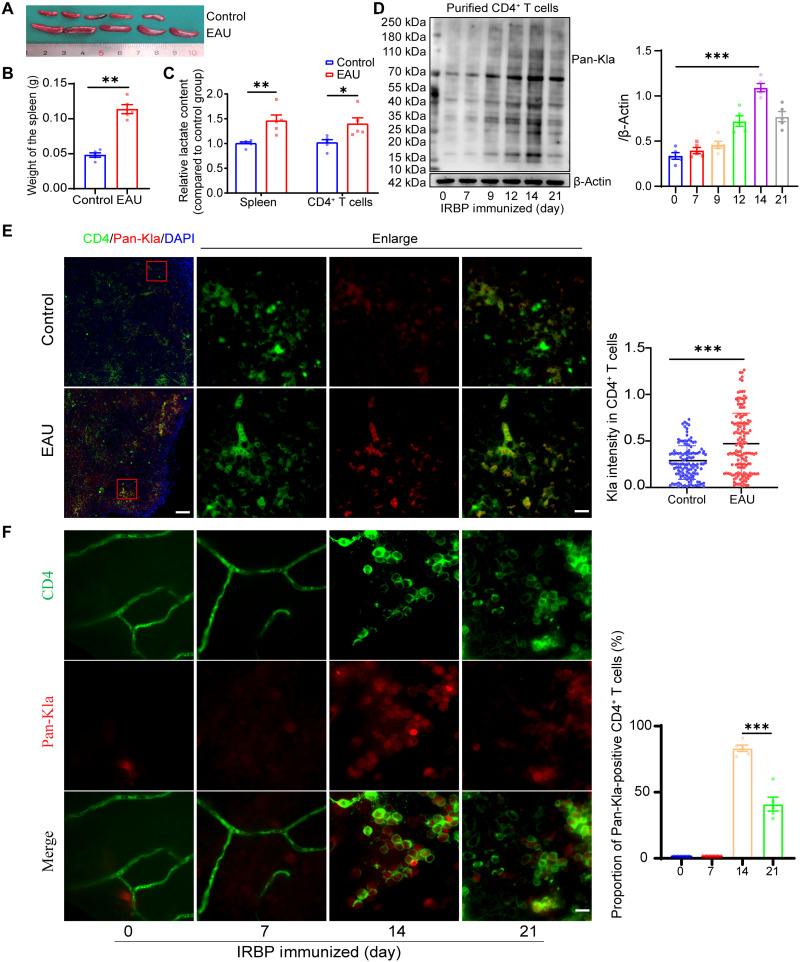
Elevated lactylation levels in CD4^+^ T cells of EAU mice. (**A** and **B**) Splenic weights of normal (control) and EAU mice (*n* = 5 mice per group; **P < 0.01 by two-tailed unpaired Student’s *t* test). (**C**) Concentrations of lactate in the spleens and CD4^+^ T cells of normal (control) and EAU mice (*n* = 5 mice per group; *P < 0.05; **P < 0.01 by two-tailed unpaired Student’s *t* test). (**D**) Lactylation levels of CD4^+^ T cells isolated from the spleen at different time points after immunization measured through Western blotting. [*n* = 5 samples per group; **P < 0.001 by one-way analysis of variance (ANOVA) and Bonferroni post hoc test]. (**E**) Representative images of CD4 costained with Pan-Kla in spleen samples [scale bars, 300 μm (left) and 40 μm (right); *n* > 100 CD4^+^ T cells from five spleens per group; four areas were randomly selected in each spleen; ***P < 0.001 by Mann-Whitney *U* test]. (**F**) Representative images of CD4 costained with Pan-Kla in retina samples (scale bar, 20 μm; *n* = 5 retinas; ***P < 0.001 by two-tailed unpaired Student’s *t* test)

Previous experimental and clinical studies demonstrated the important role of dysregulated CD4^+^ T cell differentiation in autoimmune uveitis development (*2*, *28*). An EAU mouse model was developed as previously described, and the progression of uveitis was evaluated using slit lamp photography and pathological examination (fig. S1, A and B) (*29*–*31*). Splenocytes were isolated, and the proportions of T_H_1, T_H_17, and T_reg_ cells were analyzed using flow cytometry (FCM). The gating strategy is shown in fig. S1C. The splenic weights of the EAU mice significantly increased, but no significant differences in body weight were found between groups (Fig. 1, A and B, and fig. S1D). The lactate contents in the spleens and CD4^+^ T cells were higher in the EAU mice than in the age-matched controls (Fig. 1C). Consistent with previous studies (*32*, *33*), the proportions of T_H_1, T_H_17, and T_reg_ cells obviously increased after immunization with interphotoreceptor retinoid-binding protein (IRBP) for 14 days (acute phase of EAU), and the proportions of T_H_1 and T_H_17 cells decreased on day 21 (fig. S1, E and F). CD4^+^ T cells were isolated using immunomagnetic beads (purity, >96%; verified using FCM; fig. S1G). Notably, the lactylation levels in the CD4^+^ T cells increased with EAU progression (Fig. 1D). Meanwhile, immunofluorescence staining showed hyperlactylation of CD4^+^ T cells in the spleens of the EAU mice (Fig. 1E). With the progression of uveitis, pathologic immune cells disrupted the blood-retinal barrier and triggered destructive responses in the retina. The CD4^+^ T cells that infiltrated the retina on day 14 were hyperlactylated, and the proportion of Pan-Kla–positive CD4^+^ T cells decreased on day 21 (Fig. 1F). These results indicate that lactylation is involved in the immune reactions of CD4^+^ T cells in response to pathological stimulation in autoimmune uveitis.

### Inhibition of lactylation suppressed EAU progression

**Fig. 2.**
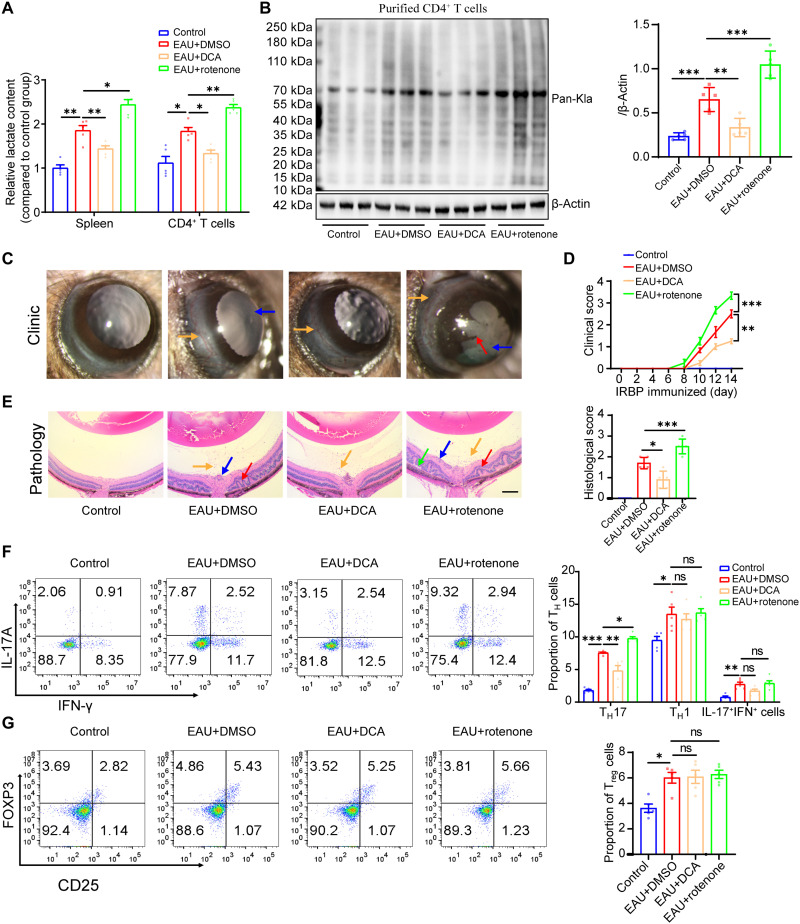
Inhibition of lactylation suppressed EAU progression. (**A**) Concentrations of lactate in the spleens and CD4^+^ T cells of normal (control) and EAU+DMSO (dimethyl sulfoxide), EAU+DCA, and EAU+rotenone groups (*n* = 5 mice per group; *P < 0.05, **P < 0.01, and ***P < 0.001 by one-way ANOVA and Dunnett’s T3 post hoc test). (**B**) Lactylation levels of CD4^+^ T cells isolated from the spleen in normal (control) and EAU+DMSO, EAU+DCA, and EAU+rotenone groups measured through Western blotting (*n* = 5 mice per group; **P < 0.01 and ***P < 0.001 by one-way ANOVA and Bonferroni post hoc test). (**C** and **D**) Anterior chamber inflammation in the control and EAU+DMSO, EAU+DCA, and EAU+rotenone groups. Clinical scores were exhibited (*n* = 5 mice per group; yellow arrows, conjunctival and/or ciliary congestion; blue arrow, anterior chamber inflammation; red arrow, iris adhesions; **P < 0.01 and ***P < 0.001 by one-way ANOVA and Bonferroni post hoc test). (**E**) Retinal histopathological images of the control and EAU+DMSO, EAU+DCA, and EAU+rotenone groups (scale bar, 100 μm; *n* = 5 mice per group; yellow arrows, infiltration of inflammatory cells; blue arrow, retinal vasculitis; red arrow, retinal folds; green arrow, retinal detachment; *P < 0.05 and **P < 0.01 by one-way ANOVA and Bonferroni post hoc test). (**F** and **G**) FCM analysis of the percentages of T_H_1, T_H_17, and T_reg_ cells in the splenocytes of the control and EAU+DMSO, EAU+DCA, and EAU+rotenone groups (*n* = 5 mice per group; ns, no significance; *P < 0.05, **P < 0.01, and ***P < 0.001 by one-way ANOVA and Bonferroni post hoc test).

The balance between glycolysis and oxidative phosphorylation affects lactate production. Considering that endogenous lactate production is a key determinant of lactylation, we analyzed whether regulating lactylation levels by modulating lactate production would affect EAU progression. The inhibitor of pyruvate dehydrogenase kinase, dichloroacetate (DCA), could shift cellular metabolism from glycolysis to oxidative phosphorylation and reduce lactate production and decrease lactylation (*17*, *19*). Experimental and clinical studies showed the prospective efficacy of DCA treatment in various therapies (*34*, *35*). In the present study, DCA treatment reduced lactate production and lactylation levels in CD4^+^ T cells (Fig. 2, A and B). It also decreased the clinical and pathological scores of the EAU mice (Fig. 2, C to E). By contrast, blocking mitochondrial metabolism with rotenone increased the lactylation of CD4^+^ T cells and aggravated the progression of EAU (Fig. 2, A to E). The proportion of T_H_17 cells in the splenocytes on day 14 decreased in the EAU+DCA group (EAU mice treated with DCA), while it increased in the EAU+rotenone group (EAU mice treated with rotenone). However, no significant differences in the proportions of T_H_1 and T_reg_ cells were found between the EAU+DCA and EAU+rotenone groups (Fig. 2, F and G). Consistent results were observed when examining the proportions of T_H_1 and T_H_17 cells in the cervical draining lymph nodes (CDLNs) of the EAU mice in response to DCA and rotenone treatments (fig. S2A). We supposed that lactylation mainly affected T_H_17 function under current conditions and that T_H_17 cells played vital roles in modulating EAU development. As previously reported, adoptively transferring IRBP-immunized T_H_17-specific cells into naïve mice can induce EAU, and suppressing T_H_17 function alone could efficiently inhibit EAU progression (*2*, *36*, *37*).

Lactate transporters (monocarboxylate transporter) are important regulators of cellular lactate content, and the redox state would affect lactate transporters (*38*). We detected the expression of lactate transporters and the level of reactive oxygen species (ROS) in induced T_H_1 and T_H_17 cells in vitro and found no significant differences between them (fig. S2, B to D). Considering that T_H_1 and T_H_17 cells are distinct producers of cytokines because of their different gene expression patterns and epigenetic characteristics (*39*), we suppose that T_H_17 fate determinant proteins that are differentially lactylated exist in this process and influence T_H_17 differentiation. Collectively, these results indicate that modulating the lactylation levels in CD4^+^ T cells can influence T_H_17 cell differentiation and EAU progression.

### Lactylation modulates T_H_17 differentiation in vitro

**Fig. 3.**
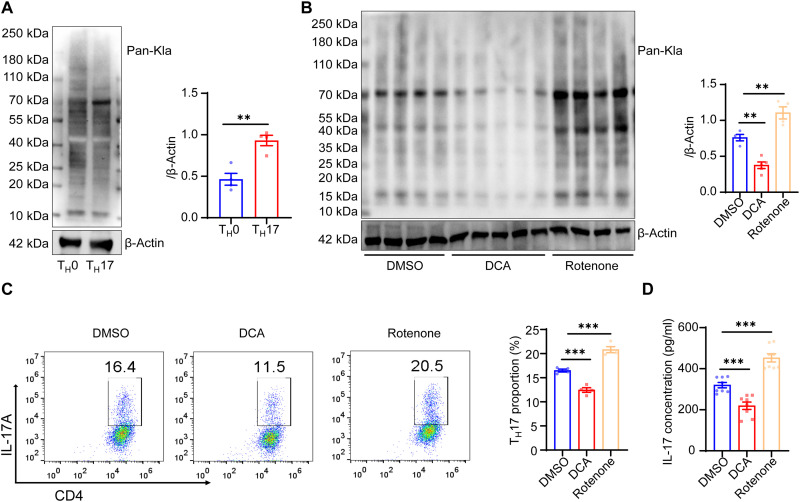
Lactylation modulates T_H_17 differentiation in vitro. (**A**) Naïve CD4^+^ T cells purified from the spleens and LNs of wild-type (WT) mice were polarized under T_H_17-inducing conditions. Lactylation levels of CD4^+^ T cells in T_H_0 and T_H_17 groups were measured through Western blotting (*n* = 4 samples per group; **P < 0.01 by two-tailed unpaired Student’s *t* test). (**B**) Lactylation levels of T_H_17 cells in the DMSO, DCA, and rotenone groups were measured through Western blotting (*n* = 4 to 5 samples per group; **P < 0.01 by one-way ANOVA and Bonferroni post hoc test). (**C**) FCM analysis of the percentage of T_H_17 cells in the DMSO, DCA, and rotenone groups (*n* = 4 samples per group; ***P < 0.001 by one-way ANOVA and Bonferroni post hoc test). (**D**) Expression levels of IL-17 in the media of the DMSO, DCA, and rotenone groups were tested using ELISA (*n* = 8 samples per group; ***P < 0.001 by one-way ANOVA and Bonferroni post hoc test).

We investigated whether lactylation regulates T_H_17 differentiation in vitro. Purified naïve CD4^+^ T cells were isolated by magnetic sorting and stimulated with anti-CD3 and CD28 antibodies alone (T_H_0 conditions) or in the presence of IL-6, TGF-β, IL-23, and IL-1β (T_H_17 conditions). After 5 days of induction, cytoplasmic IL-17 production was analyzed using FCM. We found that the lactylation levels were higher in the CD4^+^ T cells under T_H_17 conditions than in those under T_H_0 conditions (Fig. 3A). DCA treatment down-regulated the lactylation levels in the CD4^+^ T cells and suppressed the differentiation of T_H_17 cells, whereas rotenone treatment up-regulated the lactylation levels and enhanced T_H_17 differentiation (Fig. 3, B and C). Secretory IL-17 was detected using enzyme-linked immunosorbent assay (ELISA), and similar results were obtained (Fig. 3D). These results indicate that the lactylation in CD4^+^ T cells is important for T_H_17 differentiation. Previous studies indicate that abnormal proliferation of T_H_17 cells is often observed in dysregulated autoimmunity, and lactate is an important regulator of T cell proliferation (*2*, *40*). We wondered whether hyperlactylation would affect the proliferative capacity of T cells. We found that the proliferative capacity of T_H_17 cells (with high lactylation level) was higher than that of T_H_0 cells (with low lactylation level). Meanwhile, the proliferative capacities of T_H_17 cells correlated with lactylation levels in response to DCA (down-regulating lactylation) and rotenone (up-regulating lactylation) treatments (fig. S2E).

### Global view of lactylated proteins in CD4^+^ T cells

**Fig. 4.**
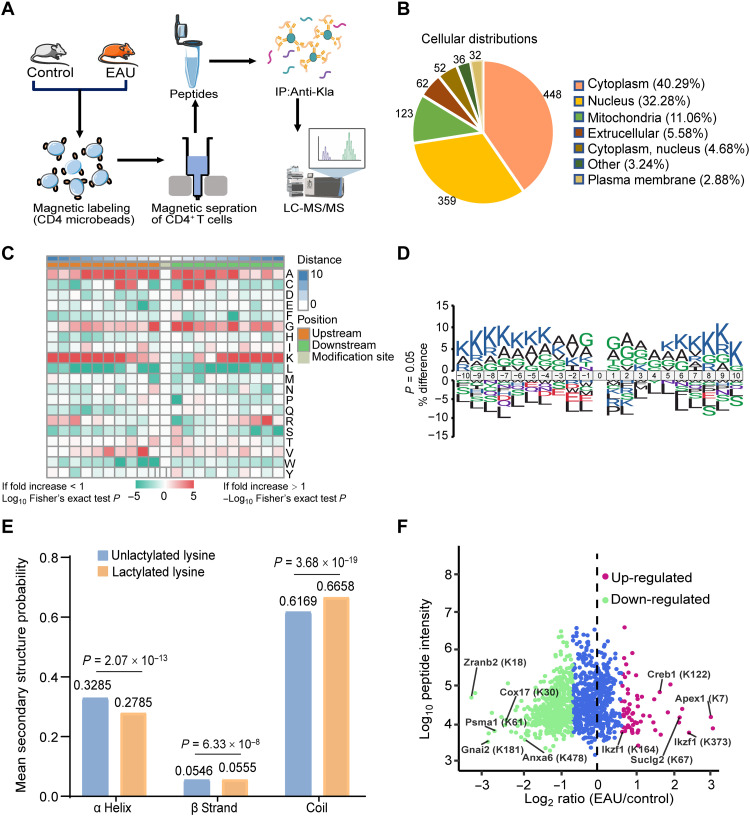
Global view of lactylated proteins in CD4^+^ T cells. (**A**) Schematic representation of experimental workflow for quantification of lactylation in normal and EAU mice CD4^+^ T cells. (**B**) Subcellular localization and classification of lactylated proteins. (**C**) Motif analysis of all identified Kla proteins. (**D**) Icelogo representation showing flanking sequence preferences for all Kla sites. (**E**) Distribution of lactylated and unlactylated lysine in structured regions of proteins. (**F**) Scatter plot showing the quantification of Kla sites in relation to peptide intensities.

We characterized the whole landscape of the lactylated proteins of CD4^+^ T cells in autoimmune uveitis. To investigate Kla substrates on normal and EAU mice CD4^+^ T cells, we used an integrated approach involving immunoaffinity enrichment and a four-dimensional mass spectrometer. A total of 1 × 10^8^ CD4^+^ T cells isolated from over 10 mice from each group were harvested and lysed. The peptides were digested with trypsin, enhanced with immobilized anti-Kla, and then subjected to liquid chromatography–tandem mass spectrometry (LC-MS/MS; Fig. 4A). We obtained 52,855 secondary spectra and 9154 available spectra for further analyses. We identified 5083 peptides in total, 2197 of which were lactylated (fig. S3A). These peptides were distributed within a reasonable range (fig. S3B). Further analysis identified 2204 lactylated sites in 751 proteins (fig. S3A). Among these lactylated proteins, 93 (12.4%) had more than six Kla sites, whereas 335 (44.6%) had one Kla site (fig. S3C). The average surface accessibility of the lactylated protein were higher (*P* = 0.001) than that of the unlactylated protein lysine residues (fig. S3D). The lactylated proteins were distributed across different subcellular localizations. A total of 359 lactylated proteins (32.28%) were identified in the nucleus; a total of 448 (40.29%) were identified in the cytoplasm, and 123 (11.06%) were identified in the mitochondria (Fig. 4B).

Thousands of protein posttranslational modification sites can be identified using large-scale modification omics. Understanding the bias of enzymes toward their substrates can help clarify the underlying biological processes responsible for these modifications. Considering that part of the biochemical preference of an enzyme for a given substrate may be determined by residues surrounding the modification site, we focused on identifying key adjacent residues that cause specific enzyme-substrate interactions of lactylation. The frequency variation of amino acids near the lactylated lysine residues is shown in Fig. 4C. Motif-X analysis identified KxAxxxxxxxK as greatly overrepresented hotspots for Kla sites (Fig. 4D and fig. S4). Structural property analysis using NetSurfP revealed that most of the lactylated sites were located in coils (67%) and helices (28%), and the remaining 5% were located in strands (Fig. 4E). Compared with the unlactylated residues, the lactylated residues showed greater preference in strands and coils, suggesting the structural preference for Kla in CD4^+^ T cells. A scatterplot displaying the quantification of the Kla sites in relation to peptide intensities is shown in Fig. 4F.

### Hyperlactylation of Ikzf1 at Lys^164^ is important for T_H_17 differentiation

**Fig. 5.**
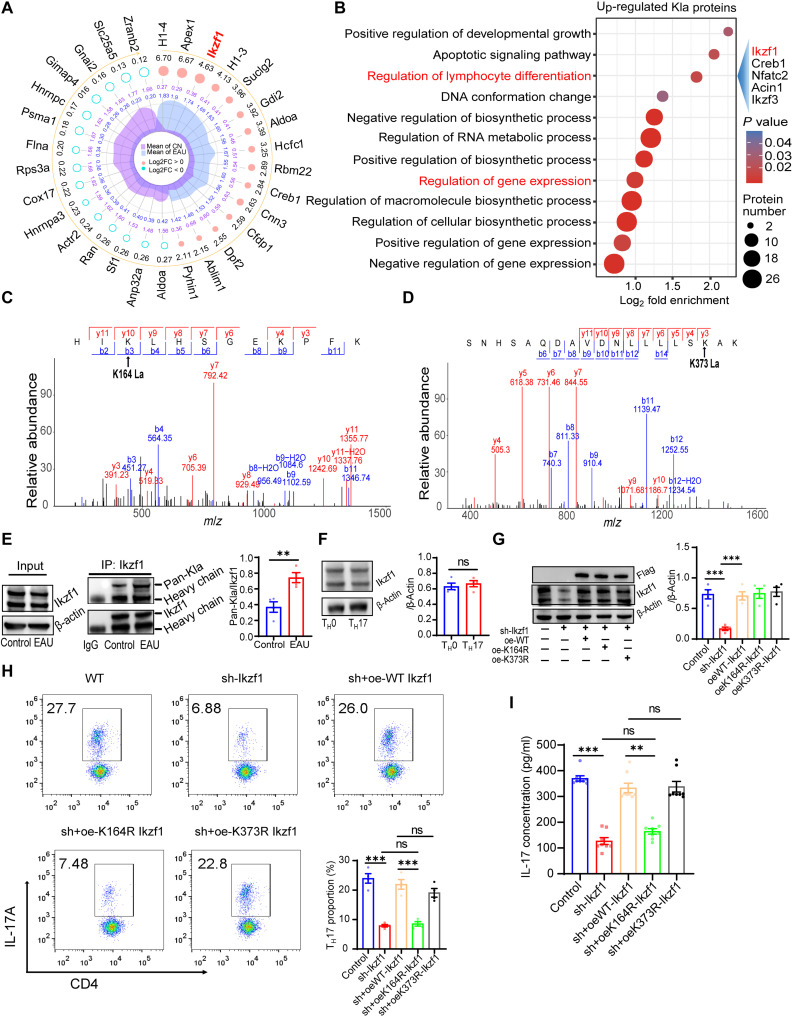
Hyperlactylation of Ikzf1 at Lys^164^ is important for T_H_17 differentiation. (**A**) Radar diagram showing the top 15 DLPs in CD4^+^ T cells of EAU mice. (**B**) Enriched GO biological processes of up-regulated Kla proteins. (**C** and **D**) MS/MS spectra of the lactylated peptides of Ikzf1 at Lys^164^ and Lys^373^. (**E**) Immunoblot after immunoprecipitation (IP) assays showing increased lactylation of Ikzf1 in CD4^+^ T cells of EAU mice (*n* = 4 samples per group; **P < 0.01 by two-tailed unpaired Student’s *t* test). (**F**) Expression of Ikzf1 in CD4^+^ T cells of T_H_0 and T_H_17 groups measured through Western blotting (*n* = 4 samples per group; ns, no significance by two-tailed unpaired Student’s *t* test). (**G**) CD4^+^ T cells were transfected with the control virus (control), only sh-Ikzf1 (sh-Ikzf1), shIkzf1+oeWT-Ikzf1 (oeWT-Ikzf1), shIkzf1+oeK164R Ikzf1 (oeK164R-Ikzf1), and shIkzf1+oe K373R Ikzf1 (oeK373R-Ikzf1) adenovirus. Expression levels of Ikzf1 in different groups were measured through Western blotting (*n* = 4 samples per group; ***P < 0.001 by one-way ANOVA and Bonferroni post hoc test). (**H**) FCM analysis of the frequency of T_H_17 cells in different groups as described in (G) (*n* = 4 samples per group; ***P < 0.001 by one-way ANOVA and Bonferroni post hoc test). (**I**) Expression levels of IL-17 in the media of corresponding groups were tested by ELISA (*n* = 8 samples per group; **P < 0.01 and ***P < 0.001 by Kruskal-Wallis test). *m*/*z*, mass/charge ratio.

We measured the variations in Kla proteins relative to total protein abundance in CD4^+^ T cells. The cutoff ratio was either above 1.5 or below 0.67 for notable Kla changes between the control and EAU CD4^+^ T cells. In total, 532 Kla sites in 279 proteins were differentially lactylated in the CD4^+^ T cells of the EAU mice. The top 15 differentially lactylated proteins (DLPs) are shown in Fig. 5A. Kyoto Encyclopedia of Genes and Genomes enrichment analysis revealed that pathways including antigen processing and presentation and glycolysis were enriched in these DLPs (fig. S5A). Gene Ontology (GO) enrichment analysis indicated that the enriched biological processes in the DLPs included the regulation of leukocyte-mediated cytotoxicity, positive regulation of cell killing, and regulation of nuclease activity (fig. S5B).

The Pan-Kla levels in the CD4^+^ T cells of the EAU mice were up-regulated, and inhibiting glycolysis suppressed EAU progression. Thus, we supposed that the up-regulated Kla proteins played important roles in CD4^+^ T cell differentiation. Most of the lactylation–up-regulated proteins were located in the nucleus and classified into the transcription category (fig. S5, C and D). GO analysis revealed that the lactylation–up-regulated proteins were enriched in several biological processes, including the regulation of lymphocyte differentiation and gene expression (Fig. 5B). Considering these results, we focused on the transcription factor Ikzf1. Ikzf1 is a member of the Ikaros transcription factor family that plays important roles in regulating lymphocyte development. Moreover, previous studies indicated that Ikzf1 is essential for T_H_17 differentiation (*23*, *41*).

In the present study, three lactylated sites were identified in Ikzf1. Among which, the lactylation levels at K164 and K373 were up-regulated by 1.52- and 4.63-fold, respectively. The MS/MS spectra including C-terminal y-ions and N-terminal b-ions of Ikzf1 are shown in Fig. 5 (C and D). We observed no significant differences in Ikzf1 expression between the CD4^+^ T cells of the control and EAU mice (Fig. 5E) and between the T_H_0 and T_H_17 cells (Fig. 5F), which is consistent with a previous study (*42*). Immunoblot after immunoprecipitation (IP) assays verified that the lactylation level in Ikzf1 was up-regulated in the CD4^+^ T cells of the EAU mice (Fig. 5E). We explored whether the lactylation of Ikzf1 would affect its role in modulating T_H_17 differentiation. Previous studies revealed that lysine (K)–to–arginine (R) mutation mimics the delactylated state of the protein (*43*, *44*). In the present study, we knocked down the endogenous Ikzf1 by using short hairpin RNA and overexpressed Flag-tagged wild-type (WT) Ikzf1, K164R mutation Ikzf1, and K373R mutation Ikzf1 in naïve CD4^+^ T cells, respectively (Fig. 5G). The cells were used for T_H_17 induction. Results showed that Ikzf1 knockdown impaired T_H_17 differentiation (Fig. 5H), which is consistent with previous reports, indicating the important role of Ikzf1 in regulating T_H_17 differentiation (*23*). WT and K373R Ikzf1 overexpression rescued T_H_17 differentiation, but a low differentiation ratio was still observed in the oe-K164R group (Fig. 5H). Consistently, ELISA results showed lower expression of IL-17 in the oe-K164R group than in the oe-WT and oe-K373R groups (Fig. 5I). These results indicate that Ikzf1 lactylation at Lys^164^ is important for T_H_17 differentiation.

### Ikzf1 K164la is up-regulated in CD4^+^ T cells of EAU mice

**Fig. 6.**
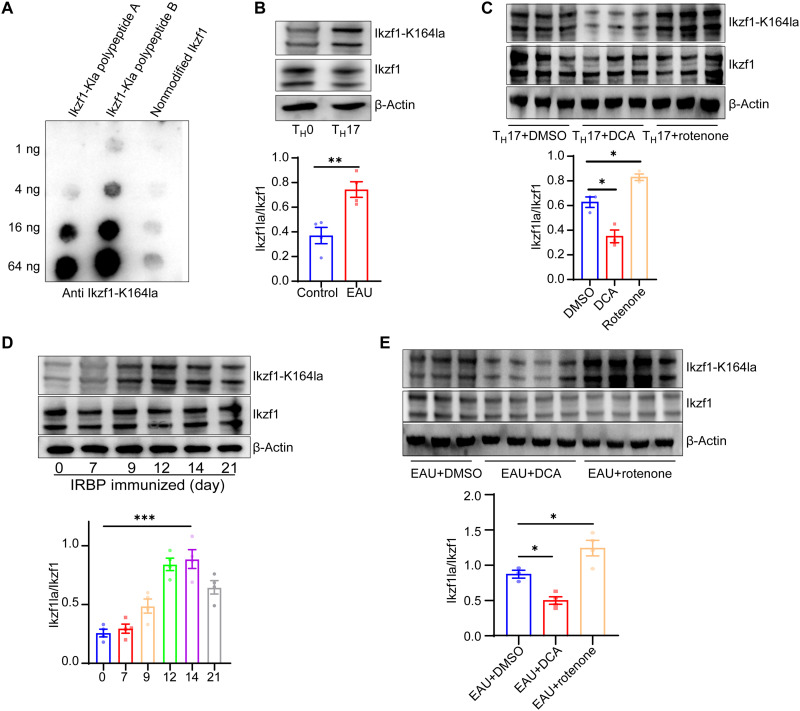
Ikzf1 K164la was up-regulated in CD4^+^ T cells of EAU mice. (**A**) Dot blot assays of Ikzf1-K164la antibody. (**B**) Ikzf1-K164la levels in T_H_0 and T_H_17 cells measured by Western blotting (*n* = 4 samples per group; **P < 0.01 by two-tailed unpaired Student’s *t* test). (**C**) Ikzf1-K164la levels in T_H_17 cells in response to DCA and rotenone treatment (*n* = 3 samples per group; *P < 0.05 by one-way ANOVA and Bonferroni post hoc test). (**D**) Ikzf1-K164la levels in CD4^+^ T cells of EAU mice at different time points (*n* = 4 samples per group; ***P < 0.001 by one-way ANOVA and Bonferroni post hoc test). (**E**) Ikzf1-K164la levels in CD4^+^ T cells of EAU mice in response to DCA and rotenone treatment (*n* = 3 to 4 samples per group; *P < 0.05 by one-way ANOVA and Bonferroni post hoc test).

To further validate the lactylation level of Ikzf1 at Lys^164^ under different conditions, we developed specialized antibodies that target modified peptides [NLLRHI-(lactyl)K-LHSGEK] of Ikzf1. Dot blot assays revealed that the constructed antibody specifically targeted the lactylated peptide of Ikzf1 and did not bind to the unlactylated Ikzf1 peptide (Fig. 6A). Using this specialized antibody, we confirmed that the lactylation levels of Ikzf1 at Lys^164^ were elevated in the CD4^+^ T cells in response to T_H_17 induction (Fig. 6B). We previously showed that rotenone treatment increased the Pan-Kla levels of CD4^+^ T cells and promoted T_H_17 differentiation. In the present study, we found that this process was accompanied by up-regulated Ikzf1 K164 lactylation, and the lactylation levels were decreased in response to DCA treatments (Fig. 6C). Meanwhile, we confirmed that Ikzf1 K164 lactylation was up-regulated with the progression of EAU and that it was regulated in response to rotenone and DCA treatment in vivo (Fig. 6, D and E). These results further indicate the pivotal role of Ikzf1 K164 lactylation in T_H_17 differentiation and autoimmune uveitis. Considering that the stemness of T_H_17 cells is implicated in the development of autoimmune diseases and it is influenced by lactate (*45*, *46*), we wondered whether the lactylation level was associated with the stemness of T_H_17 cells. We detected the Pan-Kla and Ikzf1-K164la levels in IL-1β–cultured and TGF-β–cultured T_H_17 cells, which exhibit different stemness (*47*). We found that the Pan-Kla level of the IL-1β–cultured T_H_17 cells was higher than that of the TGF-β–cultured T_H_17 cells. This result can be ascribed to the previous report that IL1-β–cultured T_H_17 cells undergo a higher level of glycolysis than TGF-β–cultured T_H_17 cells (*47*). However, no significant difference in Ikzf1 lactylation level was detected between these two groups (fig. S6). Therefore, we suppose that Ikzf1 lactylation regulates T_H_17 differentiation, but other DLPs may be involved in the differential stemness between IL-1β–cultured and TGF-β–cultured T_H_17 cells.

### CUT& Tag analysis reveals the transcriptional consequences of Ikzf1 under T_H_17 differentiation conditions

**Fig. 7.**
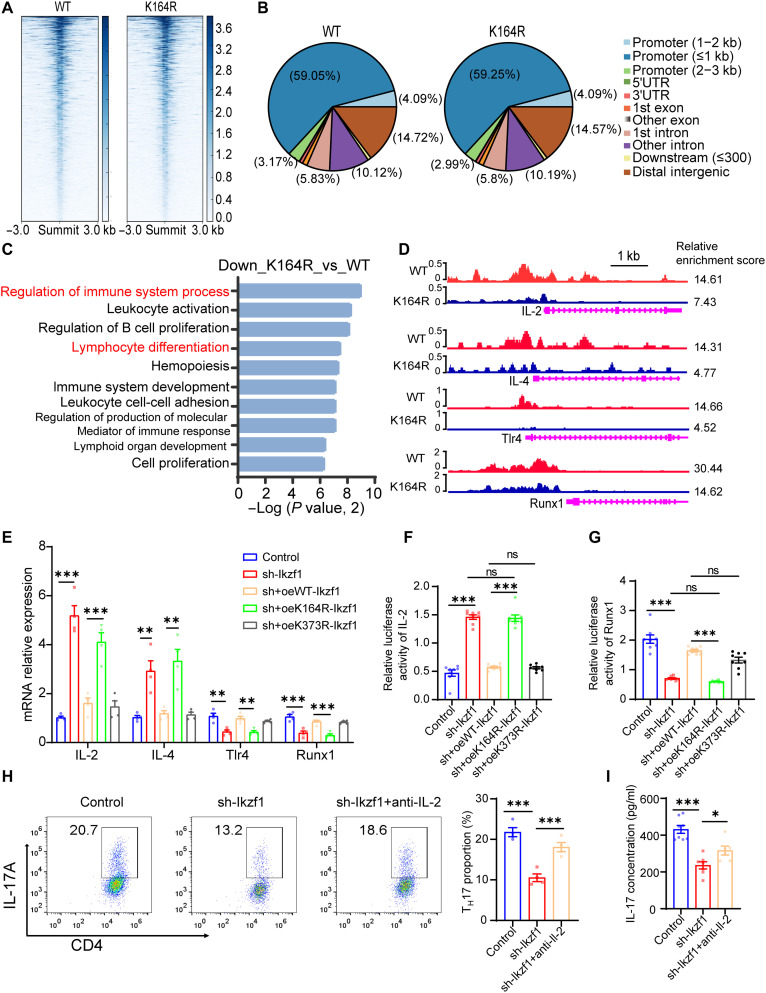
CUT& Tag analysis reveals the transcriptional consequences of Ikzf1 under T_H_17 differentiation condition. (**A**) Binding density of WT Ikzf1 was visualized by deepTools. The heatmap presents the CUT& Tag counts on the different Ikzf1 binding peaks in CD4^+^ T cells between WT and K164R groups under T_H_17 induction condition, ordered by signal strength. (**B**) Genome-wide distribution of Ikzf1 binding peaks in CD4^+^ T cells of WT and K164R groups. (**C**) GO analysis of the decreased Ikzf1 binding peaks at candidate target genes. (**D**) Genome browser tracks of CUT& Tag signal at the representative target gene loci. (**E**) mRNA expression levels of IL-2, IL-4, Tlr4, and Runx1 measured using RT-qPCR (*n* = 4 samples per group; **P < 0.01 and ***P < 0.001 by one-way ANOVA and Bonferroni post hoc test). (**F**) Luciferase activity of the IL-2 promoter–driven reporter vector was measured between the control, sh-Ikzf1, shIkzf1+oeWT-Ikzf1, shIkzf1+oeK164R-Ikzf1, and shIkzf1+oe K373R Ikzf1 groups (*n* = 8 samples per group; ***P < 0.001 by Kruskal-Wallis test). (**G**) Luciferase activity of the Runx1 promoter–driven reporter vector was measured between the control, sh-Ikzf1, shIkzf1+oeWT-Ikzf1, shIkzf1+oeK164R-Ikzf1, and shIkzf1+oe K373R Ikzf1 groups (*n* = 8 samples per group; ***P < 0.001 by one-way ANOVA and Dunnett’s T3 post hoc test). (**H**) FCM analysis of the frequency of T_H_17 cells in corresponding groups (*n* = 4 per group; ***P < 0.001 by one-way ANOVA and Bonferroni post hoc test). (**I**) Expression levels of IL-17 in the media of corresponding groups tested by ELISA (*n* = 8 per group; *P < 0.05 and ***P < 0.001 by one-way ANOVA and Bonferroni post hoc test). 5′UTR, 5′ untranslated region.

Ikzf1 is a highly conserved transcription factor (greater than 95% at the amino acid level across mice and humans) that can activate or inhibit gene transcription (fig. S7A) (*48*). Ikzf1 contains four N-terminal zinc finger (ZF) DNA binding domains and two C-terminal ZF protein-protein interaction domains (fig. S7B) (*48*). Considering that K164 is located in the second DNA binding domain of Ikzf1, we speculated that the lactylation level of Ikzf1 at Lys^164^ influences its transcription. Therefore, we carried out CUT& Tag analysis to explore possible genes modulated by Ikzf1 K164la in CD4^+^ T cells and investigate the potential functional significance of Ikzf1 K164la in T_H_17 development. Briefly, Ikzf1 knockdown naïve CD4^+^ T cells were overexpressed with oe-WT Ikzf1 or oe-K164R Ikzf1 and then used for T_H_17 induction. At 5 days after induction, the cells were harvested for CUT& Tag analysis. CUT& Tag results revealed obvious enrichment of Ikzf1 peaks in the CD4^+^ T cells under T_H_17 differentiation condition, and more than 15,000 Ikzf1 binding peaks were identified in both groups, with >60% located in the promoter sequences(≤3 kb; Fig. 7, A and B, and fig. S8A). The data from these two groups correlated well with each other (fig. S8B). The identified peaks were located evenly in the chromosomes (fig. S8C). GO analysis revealed that the down-regulated peak-related genes were enriched in multiple immune processes including lymphocyte differentiation (Fig. 7C). No significant pathways were enriched in the up-regulated peaks (*P* > 0.05; fig. S8D).

**Fig. 8.**
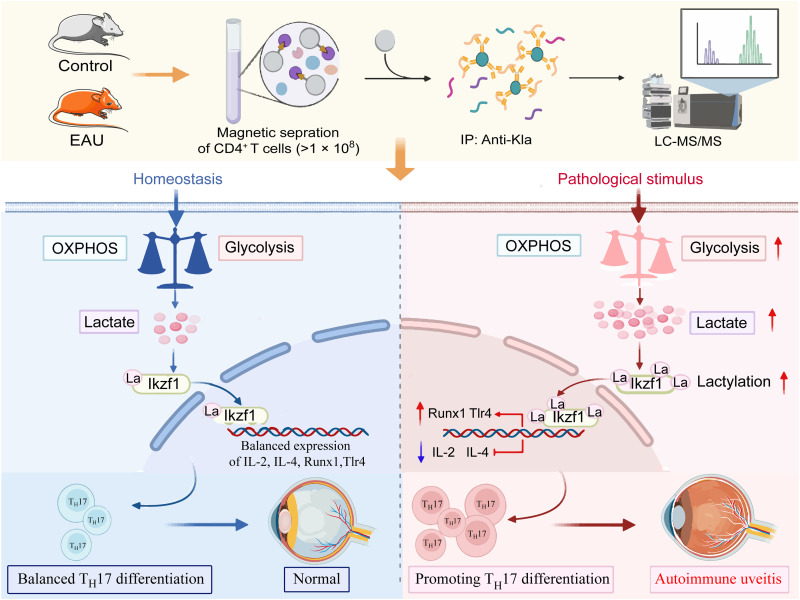
Schematic diagram of the current study. Global lactylome reveals that Ikzf1 lactylation levels are up-regulated in the CD4^+^ T cells of EAU mice. Further experiments demonstrated that Ikzf1 K164 lactylation promotes T_H_17 differentiation by regulating IL-2, IL-4, Tlr4, and Runx1 expression.

Specifically, the called peaks identified at candidate genomic loci in T_H_17 differentiation–regulated genes, including IL-2, IL-4, Tlr4, and Runx1, showed that the enrichment levels of Ikzf1 at the promoters of these genes decreased in the K164R group (Fig. 7D). Chromatin immunoprecipitation (ChIP)–quantitative polymerase chain reaction (qPCR) assays confirmed that the Ikzf1 enrichment on the IL-2, IL-4, Tlr4, and Runx1 promoters were decreased in K164R mutation cells (fig. S8E). Unexpectedly, reverse transcription qPCR (RT-qPCR) results showed that the expression levels of genes preventing T_H_17 differentiation, including IL-2 (*42*, *49*) and IL4 (*50*), were up-regulated in the sh-Ikzf1 group, whereas those of genes promoting T_H_17 differentiation, including Runx1 (*51*) and Tlr4 (*52*, *53*), were down-regulated (Fig. 7E). WT and K373R Ikzf1 overexpression inhibited IL-2 and IL-4 expression and rescued Runx1 and Tlr4 expression, but K164R Ikzf1 overexpression did not show such effects (Fig. 7E). These results further demonstrate the double transcriptional activation and inhibition abilities of Ikzf1 under the regulation of Lys^164^ lactylation. A dual-luciferase reporter assay was used to verify the differential transcriptional abilities of Ikzf1. Consistently, Ikzf1 knockdown impaired Runx1 transcription and promoted IL-2 transcription, which was reversed by WT and K373R Ikzf1overexpression but not by K164R overexpression (Fig. 7, F and G). IL-2 inhibits T_H_17 differentiation by down-regulating IL-6 receptor expression and disturbs T_H_17 gene transcription by replacing signal transducer and activator of transcription 3 (STAT3) with STAT5 on DNA sequences (*54*, *55*). Considering the correlation between Ikzf1 and IL-2 identified in our study, we hypothesized that Ikzf1 knockdown relieved the inhibition of IL-2 transcription and then impaired T_H_17 differentiation by up-regulating IL-2 expression. Therefore, we applied a recombinant anti–IL-2 antibody in Ikzf1 knockdown naïve CD4^+^ T cells. Results showed that anti–IL-2 treatment rescued T_H_17 differentiation and up-regulated IL-17 expression (Fig. 7, H and I). Collectively, these results indicate that Ikzf1 lactylation at Lys^164^ regulates T_H_17 differentiation by activating the transcription of Runx1 and Tlr4 and inhibiting the transcription of IL-2 and IL4 (Fig. 8).

## DISCUSSION

Dysregulation of T_H_17 cell differentiation plays vital roles in multiple inflammatory and autoimmune diseases, including autoimmune uveitis. In vivo neutralization of IL-17 significantly ameliorates EAU severity, and adoptively transferring IRBP-immunized T_H_17 cells into unimmunized hosts can induce EAU (*2*, *36*). In the current study, we provided evidence that lactate-derived lactylation plays an important role in regulating T_H_17 differentiation. We characterized the global lactylome of CD4^+^ T cells from normal and EAU mice. We found that the DLPs were enriched in pathways related to immune responses, including lymphocyte differentiation. Ikzf1 (K164) lactylation regulated T_H_17 differentiation by differentially modulating gene expression patterns related to T_H_17 differentiation. The above-mentioned evidence suggests that Ikzf1 lactylation is an important regulator of T_H_17 differentiation in autoimmune uveitis.

Metabolic reprogramming to glycolysis plays important roles in T_H_17 cell activation and differentiation (*11*). Glycolysis is a complex pathway that is made up of multiple processes, including the transformation of nicotinamide adenine dinucleotide (NAD^+^) and the reduced form of NAD^+^ and the production of ATP, pyruvate, lactate, and others. Various aspects of the glycolysis pathway have been proved to influence the differentiation of T_H_ cells and the progression of autoimmune diseases (*11*, *56*, *57*), but the role of lactate remains unclear. Multiple studies have implicated the cross-talk between metabolic reprogramming and posttranslational modifications (*17*, *58*–*60*). Many metabolites produced by metabolic pathways serve as substrates for posttranslational modifications. Examples of such metabolites include acetoacetyl-CoA (coenzyme A) (*58*), crotonyl-CoA (*59*), and succinate (*60*). Lactate has long been considered a waste product of aerobic glycolysis, but recent studies have implicated its important roles in regulating cellular processes by forming lactylation at certain residues (*17*, *18*). In the present study, we observed that the lactylation levels of CD^+^ T cells were up-regulated with EAU development and reduction of lactylation suppressed T_H_17 differentiation and EAU progression. Mechanistically, we found that hyperlactylation of Ikzf1 was responsible for modulating T_H_17 differentiation by regulating the expression of T_H_17-related genes. Delactylation of Ikzf1 by K164R mutation suppressed T_H_17 differentiation. Collectively, our data suggest that the progression of EAU is partly dependent on lactylation.

Previous studies indicated that exposure to lactate leads to dysfunction of T cells (*61*). For example, malignant tumor cells secrete much lactate and inhibit the proliferation and activation of T cells, which is a recognized tumor immune escape mechanism (*40*). However, it is also established that T_H_ cells are highly glycolytic in response to activation, and endogenous glycolysis is necessary for the activation, proliferation, and differentiation of T cells (*11*). T cells undergo a switch to glycolysis to promote ATP production and support T cell functions. Increased glycolysis and lactate concentrations are well-known features of inflammatory tissue microenvironment (*8*, *11*). A previous study has indicated that lactate concentrations are up-regulated in the synovia of patients with rheumatoid arthritis (*12*). Therefore, we suppose that, compared with exogenous lactate stimulation, endogenous lactate plays a different role and it may induce hyperlactylation of key proteins and then promote the function of effector T cells. We characterized the whole landscape of lactylated proteins in CD4^+^ T cells of normal and EAU mice. Analysis of the structural and subcellular location preferences of Kla sites and proteins showed that multiple proteins were differentially lactylated in the CD4^+^ T cells of the EAU mice and that these proteins were largely enriched in pathways related to immune responses, including lymphocyte differentiation. These results provide information on the DLP landscape of CD4^+^ T cells under normal and pathological conditions.

Among the DLPs, we focused on the transcription factor Ikzf1, which is essential for T_H_17 differentiation. Naïve CD4^+^ T cells cannot differentiate into T_H_17 cells without Ikzf1, and similar results were observed in our study. We detected no difference in Ikzf1 expression between the CD4^+^ T cells of the normal and EAU mice. Thus, we speculated that other factors, such as posttranslational modifications, affect the function of Ikzf1 under various conditions. Our work shows that Ikzf1 lactylation at Lys^164^ is responsible for modulating T_H_17 differentiation. Six binding domains in Ikzf1 determine its function (*48*). Notably, Lys^164^ is located in the second domain of Ikzf1 that functions in DNA binding. Therefore, we hypothesized that the lactylation status of Lys^164^ regulates the transcriptional activity of Ikzf1. Using CUT& Tag analysis, we identified a set of genes regulated by Ikzf1 lactylation. Ikzf1 is a multifunctional transcription factor that can promote or inhibit gene expression. We found that K164R mutations decreased the enrichment of Ikzf1 in the promoters of several T_H_17 differentiation–related genes, including the T_H_17-promoting genes Runx1, Tlr4, and T_H_17-inhibiting genes IL-2 and IL-4. RT-qPCR results showed that WT Ikzf1 promoted Runx1 and Tlr4 expression and inhibited IL-2 and IL-4 expression under the regulation of Lys^164^ lactylation. Notably, IL-2 is an effective cytokine that regulates T_H_17 differentiation differently in various concentrations. Low constitutive IL-2 expressed by T_H_17 cells allows T_H_17 cells to escape activation-induced cell death and promote T_H_17 function (*62*). However, high levels of IL-2 greatly inhibit T_H_17 differentiation by down-regulating IL-6 receptor expression and disturb T_H_17 gene transcription by replacing STAT3 with STAT5 on DNA sequences (*42*, *49*, *54*, *55*). Data presented in this report support that Ikzf1 hyperlactylation inhibits IL-2 expression and then promotes T_H_17 differentiation, providing mechanistic explanation for posttranslational modification–regulated IL-2 expression in autoimmune diseases.

This study has some limitations. First, our results showed that K164R mutation of naïve CD4^+^ T cells prevented T_H_17 differentiation in vitro and indicated potential mechanisms, but whether K164R mutation in vivo can inhibit T_H_17 differentiation and prevent EAU progression remains an open and interesting question. Further studies such as knocking in WT/delactylated Ikzf1 are warranted to verify our findings in vivo. In addition, other T_H_17 differentiation–related genes regulated by Ikzf1 lactylation need further exploration. Second, lactylation might be a potentially important regulator of autoimmune uveitis. We showed that multiple proteins are differentially lactylated in CD4^+^ T cells. However, the key enzymes (writers, readers, and erasers), the interaction between lactylation and other modifications, the role of glycolysis enzymes or lactate transducers, and the downstream signaling pathways remain largely unknown. Further investigations are warranted to elucidate these mechanisms.

Collectively, we explored the potential role of lactylation in T_H_17 cell differentiation and EAU progression. We characterized the lactylation landscape of CD4^+^ T cells and reported that Ikzf1 lactylation at Lys^164^ regulated T_H_17 differentiation. Furthermore, we verified the double transcriptional activation and inhibition roles of Ikzf1 in T_H_17-related genes, which are regulated by lactylation. Therefore, our study expands the scope of the Kla proteome and demonstrates previously undiscovered roles of lactylation. It also introduces Ikzf1 (K164) as a potential therapeutic target for autoimmune diseases, but its clinical application requires further investigation.

## MATERIALS AND METHODS

### Animals

Mice were obtained from the Experimental Animal Center of Chongqing Medical University (female, C57BL/6J, and 7 to 8 weeks old) and housed in a pathogen-free environment. All procedures are supported by the Ethics Committee of the First Affiliated Hospital of Chongqing Medical University (2021-613) and in compliance with Association for Research in Vision and Ophthalmology guidelines.

### EAU induction and treatment

EAU was induced as previously described (*31*). *Mycobacterium tuberculosis* strain H37Ra (40 mg; BD, 231141) and human IRBP651-670 (LAQGAYRTAVDLESLASQLT; 500 mg; Sangon) were dissolved in Freund’s adjuvant (1 ml; Sigma-Aldrich, F5881) and phosphate-buffered saline (1 ml), respectively. Then, Freund’s adjuvant and IRBP were emulsified for 1 hour in an equal volume. For the EAU model, the mice were subcutaneously injected with IRBP (500 μg) and intraperitoneally injected with 1 μg of pertussis toxin (List Biological Laboratories, Campbell, CA, USA). To ensure model success, all immunized mice underwent slit lamp examination 7 days after EAU induction and then randomized into different groups. DCA and rotenone were dissolved in 0.1% dimethyl sulfoxide (DMSO). The mice in the EAU+DCA and EAU+rotenone groups were intraperitoneally administered with DCA [200 mg/(kg·day); Rhawn] and rotenone [1.5 mg/(kg·day); Rhawn], respectively at 7, 9, 11, and 13 days after IRBP immunization. Clinical and histopathological assessments were blindly scored by two researchers according to previous reports (*63*, *64*).

### Induction of T_H_1 and T_H_17 cells in vitro

Naïve CD4^+^ T cells were magnetically sorted from the spleen and CDLNs of normal mice with a naïve CD4^+^ T Cell Isolation Kit (Stem cells, 19765). The 24-well plate was precoated with anti-mouse CD3 (2 μg/ml; BioGems, 05112-25-100) and anti-mouse CD28 (2 μg/ml; BioGems, 10312-25-100) the day before naïve CD4^+^ T cells were seeded at a density of 0.5 × 10^6^ per well. The differentiation of T_H_1 was stimulated by IL-12 (10 ng/ml; Peprotech) and IL-2 (10 ng/ml; Peprotech). The differentiation of T_H_17 cells was stimulated by adding IL-1β (20 ng/ml; Peprotech), IL-23 (20 ng/ml; BioLegend), IL-6 (25 ng/ml; Peprotech), and TGF-β (3 ng/ml; Peprotech). The cells were supplemented with fresh media on day 3. Where indicated, T cells were treated with DCA (20 mM) or rotenone (20 nM). T_H_17^TGF-β1^ [TGF-β1 (3 ng/ml) and IL-6 (25 ng/ml)] and T_H_17^IL-1β^ [IL-1β (20 ng/ml) and IL-6 (25 ng/ml)] cells were cultured as previously described (*47*).

### Adenovirus infection and plasmid transfection

The adenovirus subcloned with cDNAs of Flag-tagged Ikzf1 WT, Flag-tagged Ikzf1 K164R mutant or K373R mutant, and sh-Ikzf1 was constructed by Shanghai Sangon Biotech Co. Ltd. The adenovirus was added at a multiplicity of infection of 80, and the medium was replaced 12 hours later. The plasmids used for luciferase activity assays were acquired from Shanghai GeneChem Co. Ltd. and transfected using Lipofectamine 2000 (Invitrogen, 11668019). All protocols were carried out following the manufacturer’s instructions.

### Immunofluorescence staining

The eyeballs were shredded into flat mounts after being fixed in 4% paraformaldehyde for 2 hours. Retinal and splenic samples were blocked with 0.4% Triton X-100 and 5% goat serum for an hour and then incubated with primary antibodies at 4°C overnight. Following a meticulous washing, the samples were incubated with secondary antibody combinations for 1 hour at 37°C. Confocal microscopy (Leica, Germany) was used to capture and analyze the images. The following primary antibodies were used: CD4 (diluted 1:50; Santa Cruz Biotechnology, sc-19641) and pan-Kla (diluted 1:100; PTM-1401RM).

### Hematoxylin and eosin staining

Eyeball tissue was fixed with 10% paraformaldehyde and then wrapped in paraffin wax. The samples were sectioned at a thickness of 4 μm. After fixation and dehydration, the sections were stained with hematoxylin and eosin.

### Quantification of lactate levels

The amount of lactate present in the splenic tissue and cells was assessed using an LA Content Assay Kit (Solarbio, BC2235). Briefly, lactate was extracted from same amounts of samples from various groups using extracting solutions A and B. Then, the supernatant was added with reaction solution and color developing solution. The lactate concentrations were measured by detecting the absorbance at 570 nm on Thermo Fisher Scientific Varioskan LUX Microplate reader.

### Real-time qPCR

Total RNA was extracted using an RNA extraction kit (Accurate Biology, AG21023) and then reverse transcribed using RT Master Mix for qPCR (MCE, HY-K0510). Real-time qPCR was carried out in a 20-μl system using the SYBR Green qPCR Master Mix (MCE, HY-K0501) and ABI Prism 7500 machine (Applied Biosystems, CA, USA). β-Actin was used as the internal control, and the results were calculated using the ΔΔ*C*_t_ method. The following gene-specific primers were used for RT-qPCR: IL-2, F-5′TGAGCAGGATGGAGAATTACAGG and R-GTCCAAGTTCATCTTCTAGGCAC3′; IL-4, F-5′GGTCTCAACCCCCAGCTAGT and R-GCCGATGATCTCTCTCAAGTGAT3′; Tlrr4, F-5′ATGGCATGGCTTACACCACC and R-GAGGCCAATTTTGTCTCCACA3′; and Runx1, F-5′GATGGCACTCTGGTCACCG and R-GCCGCTCGGAAAAGGACAA3′.

### Western blotting

Cell lysates were prepared using radioimmunoprecipitation assay lysis buffer (MCE, HY-K1001). Equal amounts of lysates (15 μg) were separated using 4 to 20% polyacrylamide electrophoresis gel and transferred onto polyvinylidene difluoride membranes (Millipore, MA, USA). After being blocked with 5% skim milk, the membranes were incubated with primary antibodies at 4°C overnight and secondary antibodies for 1 hour at 37°C. Signals detected using the ECL kit (K-12094-D50, Advansta, CA, USA) were quantified using ImageJ software and normalized to β-actin levels. The following primary antibodies were used: Pan-Kla (diluted 1:1000; PTM-1401RM), Ikzf1-K164la (PTM), Ikzf1 (diluted 1:1000; 14859, CST), β-actin (diluted 1:3000; Proteintech, 20536-1-AP), and FLAG (diluted 1:1000; 14793, CST).

### Immunoprecipitation

IP was performed using an IP kit (Abcam, ab206996). Lysates (1 mg) were incubated overnight with 4 μg of primary antibodies at 4°C with continual rotation and then incubated with 50 μl of Protein A or G Agarose beads for 4 hours. After extensive washing, the precipitated proteins were removed from the beads by resuspending in 2× SDS loading buffer and boiling for 5 min. Western blotting was performed to assess the lysates.

### ChIP assay

ChIP assays were performed with a SimpleChIP Plus Enzymatic Chromatin IP Kit (CST, 9004) in accordance with the manufacturer’s instructions. Cells at a density of 4 × 10^6^ were used for each sample. After fixation and dissociation, chromatin DNA was sheared into fragments using micrococcal nuclease and then incubated with anti-Ikzf1 (CST, 14859) or anti-IgG (CST, 3900) antibodies and Protein G Agarose beads overnight at 4°C. Then, the DNA was purified for further experiments.

### Luciferase activity assays

The promoter regions of IL-2 and Runx1 were cloned into a luciferase reporter vector and then transfected into CD4^+^ T cells together with plasmids targeting Ikzf1. Luciferase activity was measured using a dual-luciferase reporter gene assay kit (Promega, E2920). *Renilla* luciferase activity was used to normalize reporter gene activity.

### Enzyme-linked immunosorbent assay

Naïve CD4^+^ T cells were seeded in 24-well plates at a density of 5 × 10^5^/ml and induced T_H_17 differentiation for 5 days. The concentrations of IL-17 in the culture media of distinct groups were measured using a mouse IL-17 ELISA Kit (R&D Systems, M1700) in accordance with the manufacturer’s instructions.

### Flow cytometry

For IL-17 and IFN-γ staining, cells were restimulated with Cell Activation Cocktail (Brefeldin A; BioLegend, 423304) for 6 hours at 37°C.The harvested cells were stained with surface marker with fluorescein isothiocyanate anti-mouse CD4 antibody (BioLegend, 100406), fixed with fixation buffer (BioLegend, 420801), and then permeabilized (BioLegend, 421002). The cells were stained with antibodies for 40 min at 4°C. The following antibodies were used: PE anti-mouse IL-17A (Biolegend, 506904), APC anti-mouse IFN-γ (Biolegend, 505810), PE anti-mouse FOXP3 (Biolegend, 320007), and APC anti-mouse CD25 (Biolegend, 101910). The stained cells were analyzed using Thermo Fisher Scientific Attune NxT flow cytometer, and the data were processed using FlowJo software (FlowJo Co., Ashland, OR, USA). Proliferation and ROS assays were conducted through flow cytometry using EdU Cell Proliferation Kit (Beyotime, C0071S) and ROS Assay Kit (Beyotime, S0033S), respectively, in accordance with the manufacturer’s instructions.

### Pan-Kla–based PTM enrichment

Cells were sonicated using an ultrasonic processor in lysis buffer. The protein solution was digested twice with trypsin. Tryptic peptides were incubated with Pan-Kla antibody and prewashed beads (PTM-1404, PTM Bio) at 4°C overnight to enrich Kla-modified peptides. The bound peptides were eluted using trifluoroacetic acid (0.1%). After being vacuum-dried and desalted using C18 ZipTips (Millipore), the samples were used for further analyses.

### LC-MS/MS analysis and database search

LC-MS/MS analysis was conducted with the assistance of Jingjie PTM Biolabs (Hangzhou, China). Tryptic peptides were separated using a nanoElute HPLC system (Bruker Daltonics). The peptides were exposed to a capillary source before being subjected to MS using a timsTOF Pro (Bruker Daltonics). The MaxQuant search engine (v.1.6.15.0) was used to analyze MS/MS data. The relative quantitative values of the altered peptides were determined by centralizing the signal intensity levels across samples. All quantified Kla peptide ratios were adjusted in accordance with their respective protein expression levels.

### CUT& Tag

CUT& Tag was performed using the Hyperactive Universal CUT& Tag Assay Kit for Illumina (Vazyme, TD903-01) in accordance with the manufacturer’s instructions. Briefly, CD4^+^ T cells were gathered and bound to beads coated with concanavalin A, permeabilized by digitonin, and then incubated with Ikzf1 antibodies (CST, 14859). pA-Tn5 transposase was then incubated with the samples. The DNA was extracted, amplified, and purified following transposon activation and tagmentation to create a library and then analyzed on an Illumina NovaSeq 150PE platform.

### Statistical analysis

Data are presented as means ± SEM and analyzed using SPSS 20.0. The numbers in the legends represent independent biological replicates. Comparisons between two groups were performed using an unpaired Student’s *t* test or Mann-Whitney *U* test according to its normality. One-way analysis of variance (ANOVA) or Kruskal-Wallis test was applied to multiple groups as indicated (*P < 0.05, **P < 0.01, and ***P < 0.001).
