# Supplementary material for: Global lactylome reveals lactylation-dependent mechanisms underlying TH17 differentiation in experimental autoimmune uveitis
Source: Sci Adv. 2023 Oct 18;9(42):eadh4655. doi: 10.1126/sciadv.adh4655 (PMC10584346; doi:10.1126/sciadv.adh4655)
Supplement: Supplementary file 1 — Figs. S1 to S8 [file sciadv.adh4655_sm.pdf]

Science Advances
AAAS

Supplementary Materials for

Global lactylome reveals lactylation-dependent mechanisms underlying T_H17 differentiation in experimental autoimmune uveitis

Wei Fan *et al.*

Corresponding author: Shengping Hou, sphou828@163.com

Sci. Adv. **9**, eadh4655 (2023)
DOI: 10.1126/sciadv.adh4655

This PDF file includes:

Figs. S1 to S8

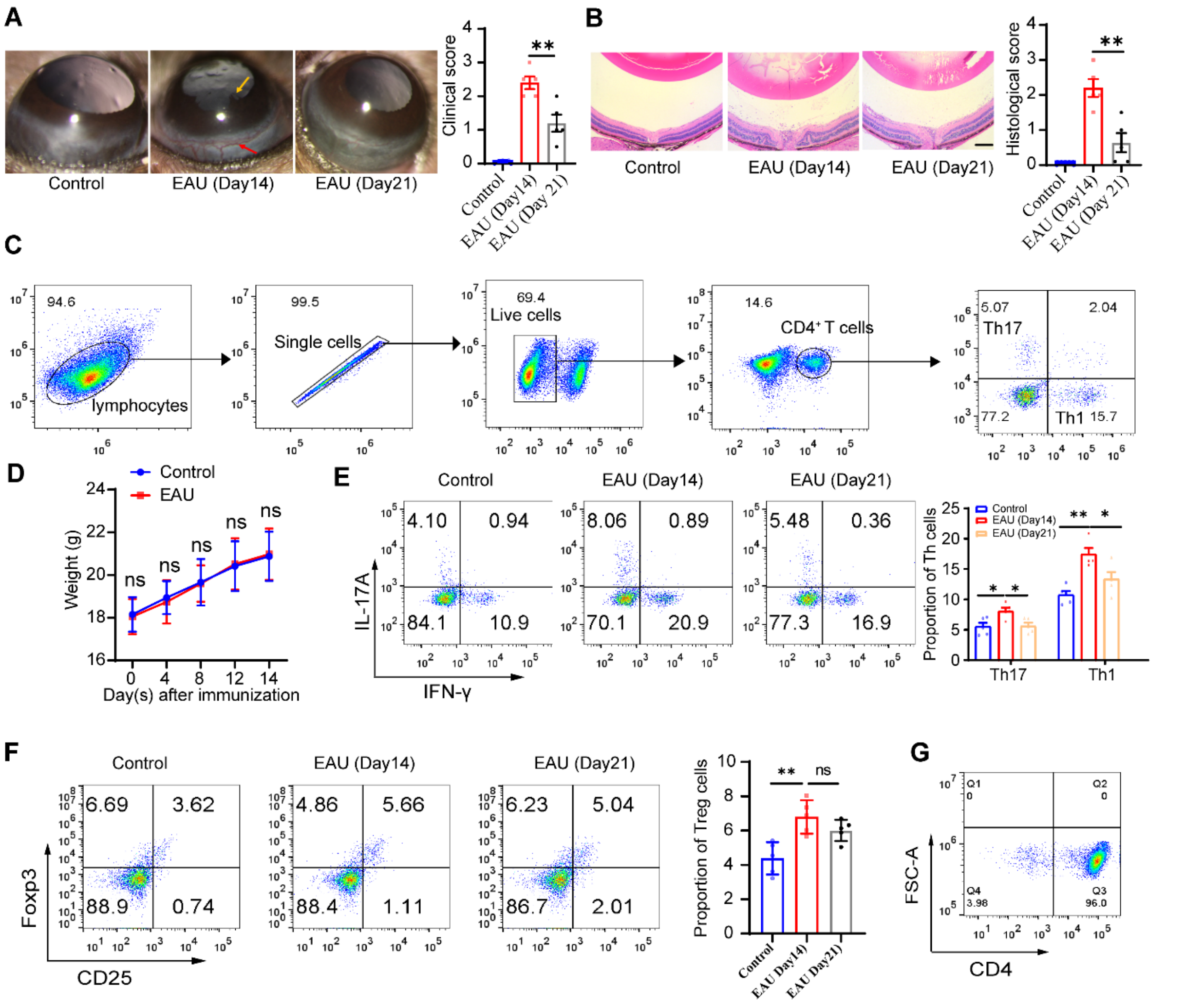

Fig. S1. Development of experimental autoimmune uveitis

(A) Anterior chamber inflammation in the control and EAU mice. (n=5 mice per group; yellow arrows, iris adhesions; red arrow, conjunctival and/or ciliary congestion; **p<0.01 by two-tailed unpaired Student's t test).

(B) Retinal histopathological images of the control and EAU mice (Scale bar, 100 µm; n=5 mice per group; **p<0.01 by two-tailed unpaired Student's t test).

(C) Gating strategy for flow cytometry.

(D) Body weight of normal (control) and EAU mice at different time points after IRBP immunization. (n=5 mice per group; ns-no significance by two-tailed unpaired Student's t test)

(E-F) FCM analysis of percentages of Th1, Th17, and Treg cells in the splenocytes of the control and EAU mice (*p<0.05; **p<0.01 by one-way ANOVA, Bonferroni post-hoc test).

(G) FCM analysis was used to verify the purity of $CD4^+$ T cells isolated from spleen.

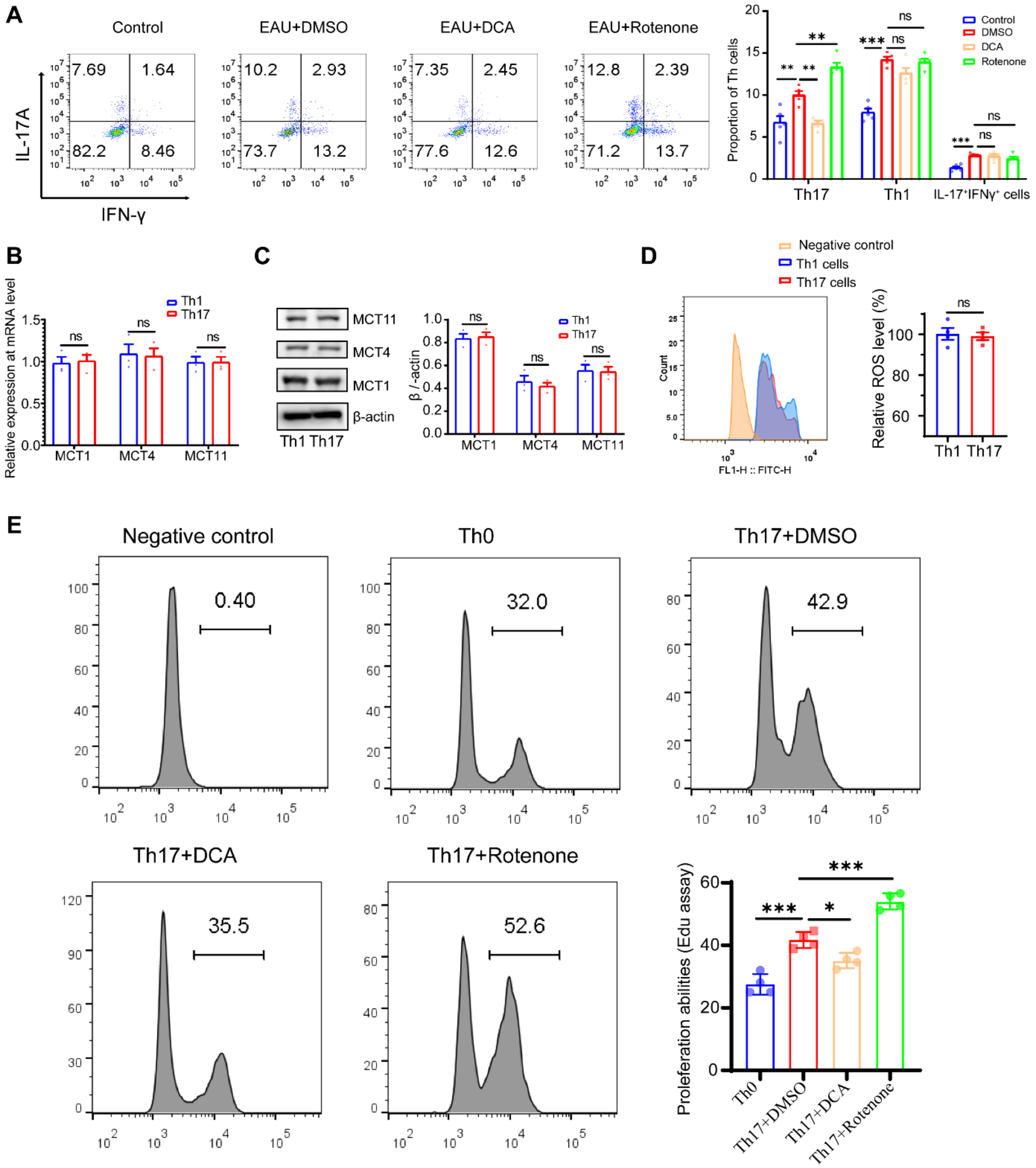

Fig. S2. Lactylation modulates Th17 differentiation.

(A) FCM analysis of the percentages of Th1, Th17 cells in the CDLNs of control and EAU mice in response to DCA and rotenone treatment (**$p<0.01$; ***$p<0.001$ by one-way ANOVA, Bonferroni post-hoc test).

(B) Expression of MCTs in Th1 and Th17 cells at mRNA level (n=3 samples per group; ns-no significance by two-tailed unpaired Student's t test).

(C) Expression of MCTs in Th1 and Th17 cells at protein level (n=3 samples per group; ns-no significance by two-tailed unpaired Student's t test).

(D) ROS levels in Th1 and Th17 cells measured by FCM (n=4 samples per group; ns-no significance by two-tailed unpaired Student's t test).

(E) Proliferative capacity of Th1 and Th17 cells measured by EdU assays (*$p<0.05$; ***$p<0.001$ by one-way ANOVA, Bonferroni post-hoc test).

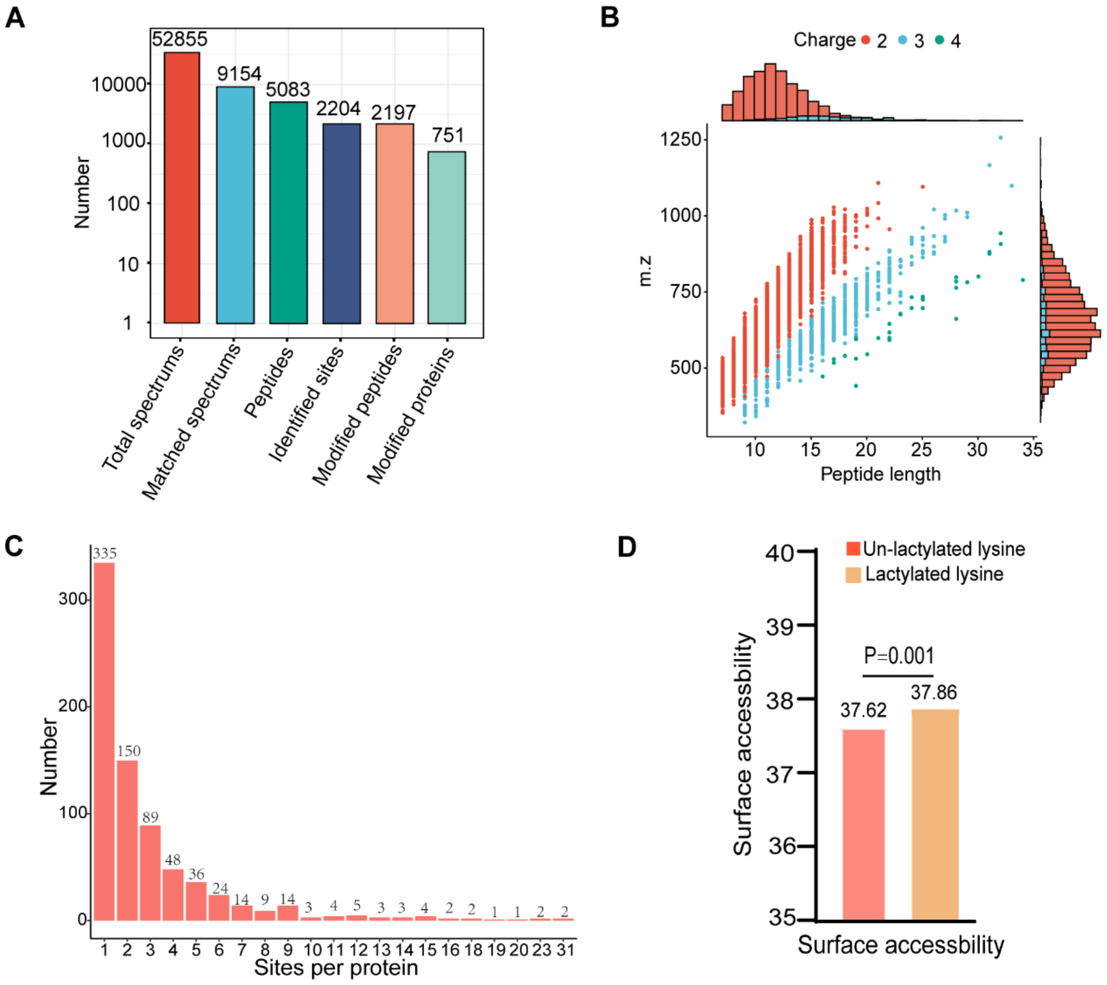

Fig. S3. Global view of lactylated proteins in CD4 T cells

(A) Details of the identified peptides and proteins.

(B) Distribution of the length of the identified peptides.

(C) Histogram showing the distribution of quantifiable Kla sites of the identified Kla proteins.

(D) The average surface accessibility of lactylated and unlactylated protein lysine residues.

Motif Logo	Motif	Motif Score	Foreground		Background		Fold Increase
			Matches	Size	Matches	Size	
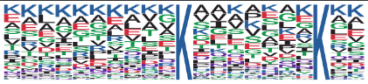	xxxxxxxxxx_K_xxxxxx xKxx	16.00	280	2201	38554	518092	1.7
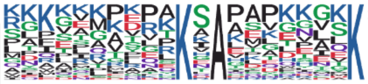	xxxxxxxxxx_K_xAxxxx xxxK	22.25	38	1921	2094	479538	4.5
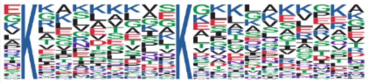	xKxxxxxxxx_K_xxxxx xxxxx	12.55	226	1883	34830	477444	1.6
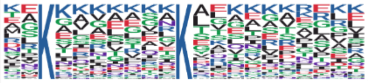	xxKxxxxxxx_K_xxxxx xxxxx	9.86	188	1657	31140	442614	1.6
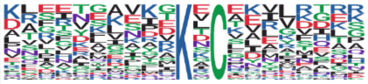	xxxxxxxxxx_K_xCxxxx xxxx	9.48	72	1469	8880	411474	2.3
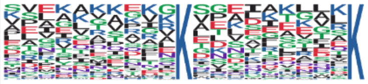	xxxxxxxxxx_K_xxxxxx xxxK	8.01	146	1397	25892	402594	1.6
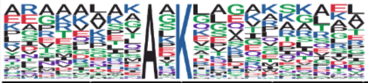	xxxxxxxxAx_K_xxxxxx xxxx	6.28	126	1251	24125	376702	1.6

Fig. S4. Sequence logos analysis of the lactylated peptides. Sequence motif logos showing the lactylation sites detected in proteins and the position-specific amino acids' composition surrounding the lactylation sites.

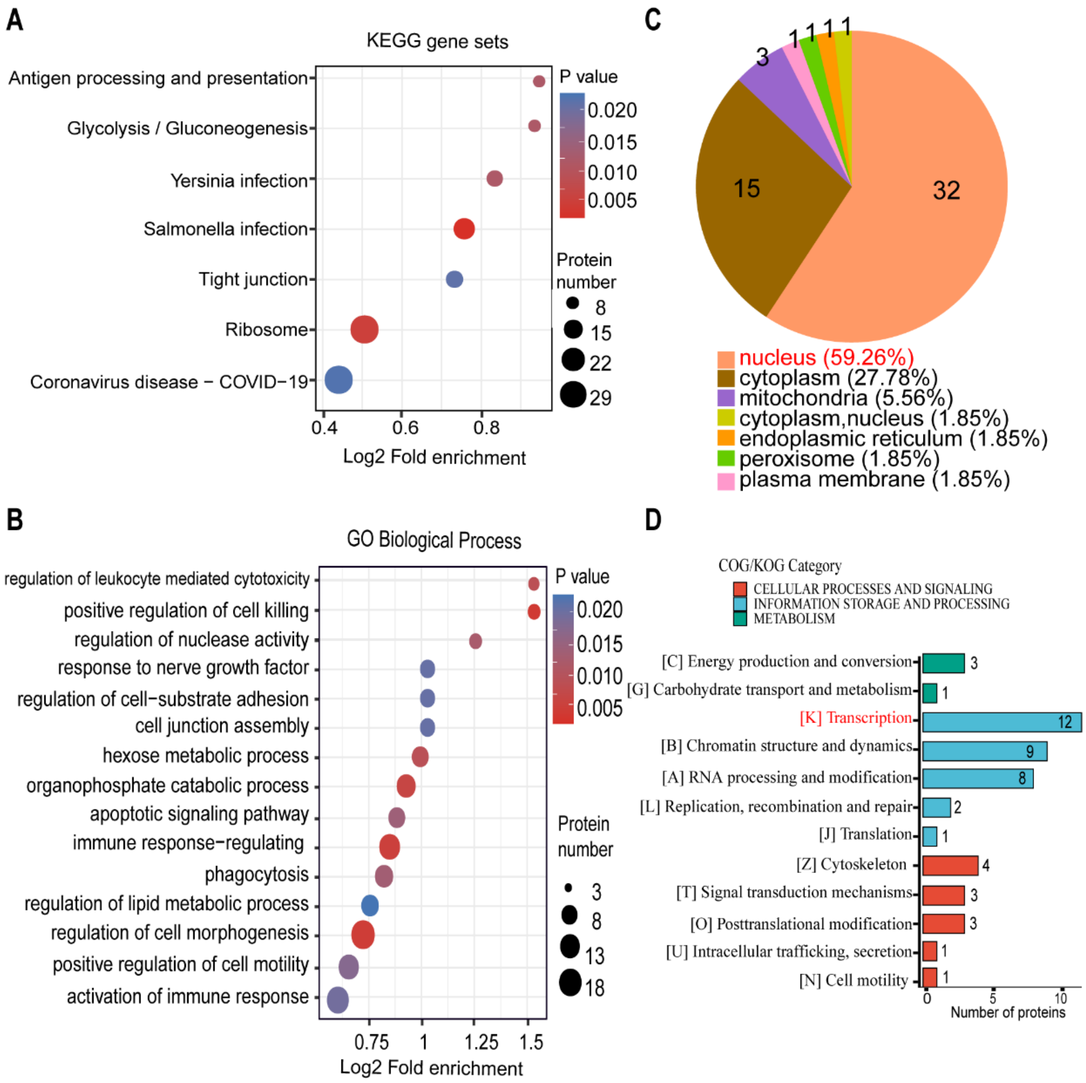

Fig. S5. Quantitative analysis of Kla proteome in normal and EAU CD4 T cells

(A) KEGG pathways enriched in differentially lactylated proteins.

(B) GO Biological processes enriched in differentially lactylated proteins.

(C) Subcellular localization and classification of the upregulated lactylated proteins.

(D) COG/KOG category of the upregulated lactylated proteins.

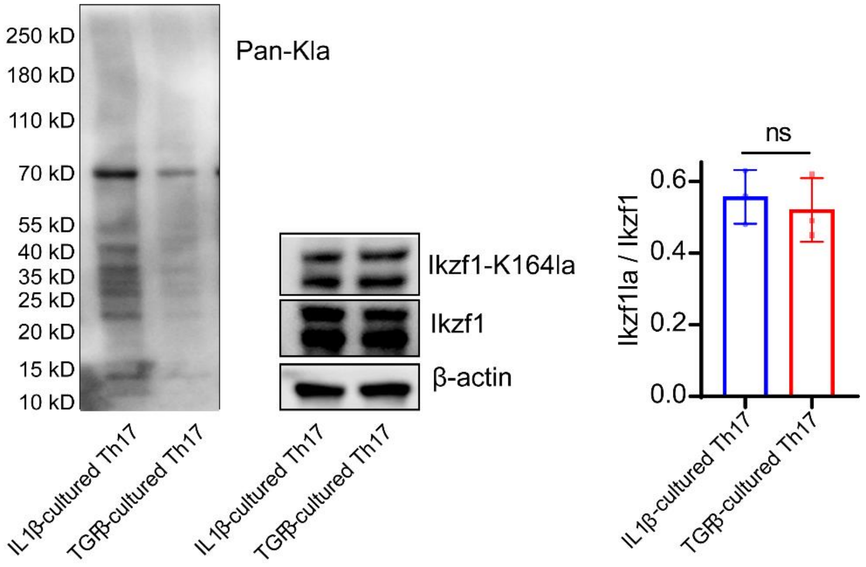

Fig S6. Pan-Kla and Ikzf1-K164la levels of IL1β-cultured and TGFβ-cultured Th17 cells (n=3 samples per group; ns-no significance by two-tailed unpaired Student's t test).

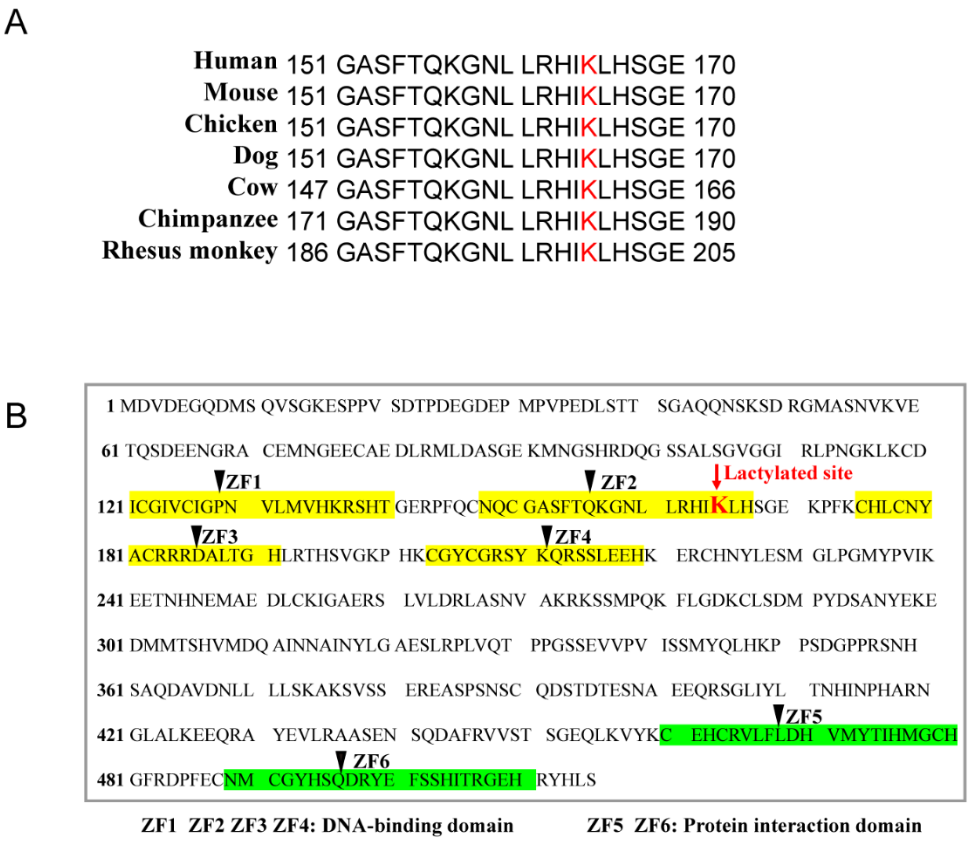

Fig. S7. Characteristics of Ikzf1

(A) Ikzf1 is a highly conserved protein. The peptide containing lactylated lysine of IKZF1 is conserved in human, mouse, chimpanzee, Rhesus monkey, dog, cow, and chicken.

(B) Ikzf1 contains four N-terminal zinc finger (ZF) DNA-binding domains and two C-terminal ZF protein-protein interaction domains.

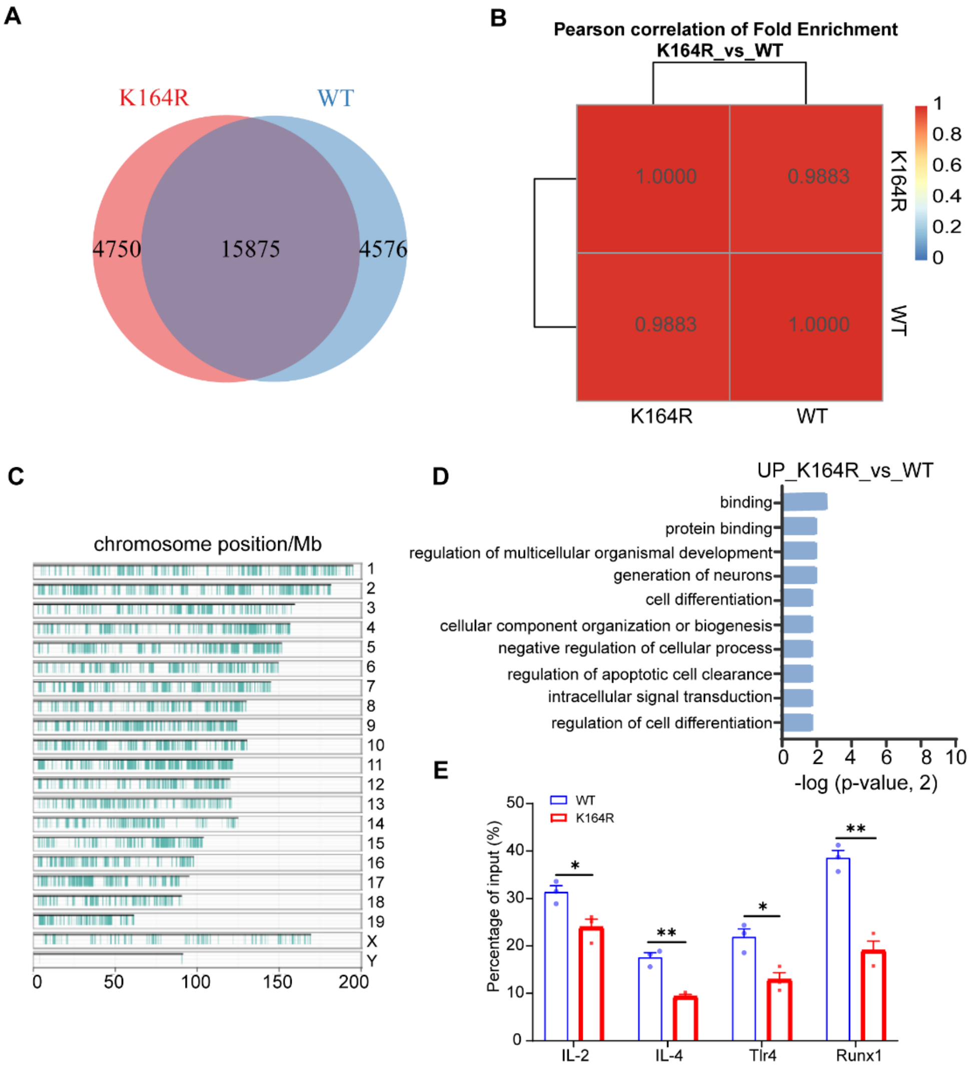

Fig. S8. Genome-wide analysis of the transcriptional consequences of Ikzf1 under Th17 differentiation condition

(A) The number of peaks identified in WT and K164R groups.

(B) Pearson correlation of fold enrichment between two groups.

(C) Location of identified peaks in chromosomes of mouse.

(D) GO analysis of the upregulated Ikzf1 binding peaks at candidate target gene.

(E) ChIP-qPCR analysis of Ikzf1 binding at the promoters of indicated genes (n=3 samples per group; *p<0.05, **p<0.01 by two-tailed unpaired Student’s t test).
